# Successful Use of Remimazolam in a Dialysis Patient With End‐Stage Renal Disease and a History of Cardiac Arrest Following Induction of Propofol Anesthesia: A Case Report

**DOI:** 10.1155/cria/7052417

**Published:** 2025-10-05

**Authors:** Toshihiro Takeda, Koji Okano, Yuichi Ogino

**Affiliations:** ^1^ Department of Anesthesia and Pain Medicine, Kagawa University Hospital, 1750-1 Ikenobe Kita-gun, Miki, 761-0793, Kagawa, Japan

**Keywords:** cardiac arrest, dialysis, end-stage renal disease, general anesthesia, motor evoked potential, remimazolam

## Abstract

**Background:**

Remimazolam, an ultra‐short‐acting benzodiazepine, has minimal hemodynamic impact compared with propofol, making it a potentially safer alternative for patients at high cardiovascular risk. This report highlights the anesthetic management of a hemodialysis patient with end‐stage renal disease who had a prior cardiac arrest induced by propofol–remifentanil anesthesia and subsequently underwent lumbar laminectomy with remimazolam.

**Case Presentation:**

A 69‐year‐old male with paroxysmal atrial fibrillation, diabetes mellitus, and dialysis‐dependent renal failure required lumbar laminectomy. During the first anesthesia attempt, induction with propofol and remifentanil led to pulseless electrical activity, necessitating resuscitation and surgery cancellation. A second attempt was planned using remimazolam for anesthesia induction and maintenance. With careful perioperative fluid management, remimazolam facilitated stable hemodynamics, and the procedure was completed uneventfully.

**Conclusion:**

This report demonstrates that remimazolam effectively stabilizes intraoperative hemodynamics and highlights its potential as a safe and viable anesthetic option for high‐risk patients with severe comorbidities, including prior cardiac arrest.

## 1. Background

Remimazolam, an ultra‐short‐acting benzodiazepine, has rapid onset and offset of effects. Its ester bonds are rapidly hydrolyzed by carboxylesterase in the liver, forming metabolites with minimal benzodiazepine receptor affinity (approximately 1/400th of remimazolam) [1]. With low levels of in vivo pharmacological activity, remimazolam has a remarkably short context‐sensitive half‐time [2]. Furthermore, as its metabolites are primarily excreted via the kidneys, they may accumulate in patients with impaired renal function. However, given their extremely low benzodiazepine receptor activity, remimazolam is considered a suitable agent even for patients with end‐stage renal disease (ESRD) [3, 4]. In addition to its rapid metabolism, remimazolam is notable for its minimal hemodynamic impact compared with other general anesthetics, especially propofol [5–7].

We administered anesthesia using remimazolam for lumbar laminectomy in a dialysis patient with ESRD and a history of cardiac arrest following the induction of general anesthesia with propofol and remifentanil, achieving stable hemodyamics.

## 2. Case Presentation

A 69‐year‐old male (height: 166.8 cm, weight: 70 kg, and BMI: 25.2 kg/m^2^) was scheduled to undergo laminectomy for lumbar spinal canal stenosis. His medical history included paroxysmal atrial fibrillation (PAF) for 20 years, diabetes mellitus (DM) for 15 years, and ESRD caused by DM. He was receiving warfarin and bisoprolol fumarate to treat PAF and hypertension, pregabalin for low back pain, mitiglinide for DM, and a daily subcutaneous injection of 15 units of a rapid‐acting insulin analog.

He had been on hemodialysis for 2 years. Notably, he frequently experienced significant drops in blood pressure during dialysis sessions, which often necessitated the elevation of his lower limbs. The ultrafiltration volume per session was typically 6 L. However, severe hypotension often prevented the complete removal of the scheduled volume, leading to the early termination of dialysis, a condition referred to as “dialysis difficulty.”

Laboratory tests revealed severely impaired kidney function, with serum creatinine levels of 12.5 mg/dL, blood urea nitrogen of 45.5 mg/dL, and serum potassium levels of 4.7 mmol/L. The patient had a 15‐year history of DM, which was complicated by ESRD. However, no peripheral neuropathy or ocular complications (such as cataracts or glaucoma) had been identified. Although the patient was receiving insulin therapy, preoperative laboratory tests revealed poor glycemic control, with an HbA1c level of 7.6%. No other abnormalities related to the liver, hemostasis, and coagulation function were detected.

Upon ultrasoundcardiography (UCG), the left ventricle (LV) exhibited diffuse mild hypokinesis with a left ventricular ejection fraction (EF) of 48%, indicating mildly reduced contractility. However, only mild aortic valve calcification was observed, and there was no significant valvular disease. The left atrial volume index (LAVI) was slightly elevated at 38.7, reflecting left atrial enlargement associated with AF. There was no evidence of pulmonary hypertension.

### 2.1. First Anesthesia Progress (Figure 1)

The patient had long‐standing DM complicated by ESRD requiring hemodialysis and was, therefore, considered to be at high risk of circulatory collapse during the induction of general anesthesia. However, preoperative UCG assessment revealed only a mild reduction in cardiac contractility, and no evidence of peripheral neuropathy and other organ dysfunction was observed. Based on these findings, we determined that general anesthesia with propofol could be performed safely under careful induction with concomitant use of vasopressors. In addition, because the surgeon strongly requested intraoperative motor evoked potential (MEP) monitoring, we selected propofol as the primary anesthetic agent, given its reported minimal interference with MEP compared with benzodiazepines and volatile anesthetics [8].

**Figure 1:**
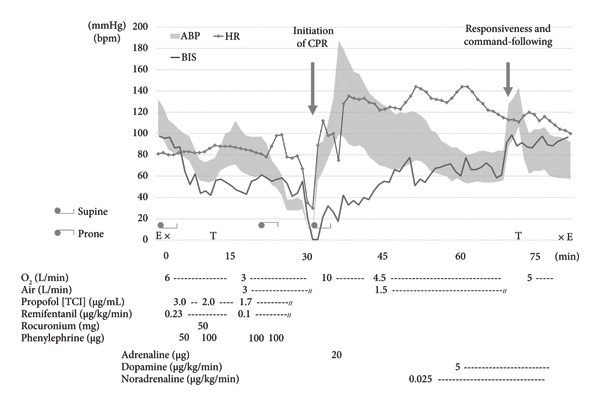
First anesthesia progress. ABP: arterial blood pressure, HR: heart rate, BIS: bispectral index, E: admission to or discharge from the operating room, X: start or end of anesthesia, T: tracheal intubation or extubation.

After entering the operating room without anesthetic premedication, the patient underwent catheter insertion into the right radial artery, followed by the invasive arterial blood pressure (ABP) monitoring (Figure 2(a)). Following confirmation by electrocardiography (ECG), percutaneous oxygen saturation (SpO_2_) and bispectral index (BIS) monitoring, general anesthesia was induced using propofol (target‐controlled infusion [TCI]), remifentanil, and rocuronium. After tracheal intubation, the systolic blood pressure (sBP) dropped to 65 mmHg (Figure 2(b)). Following the intermittent administration of phenylephrine, sBP was restored to ≥ 100 mmHg (Figure 2(c)). Since hemodynamic suppression due to anesthetic administration was a concern, TCI of propofol was reduced to 1.7 μg/mL to maintain a BIS value of approximately 50–60, and the remifentanil infusion rate was decreased to 0.1 μg/kg/min. After confirming the stability of both sedation level and hemodynamics, the patient was repositioned to the prone position.

Figure 2Wave forms during the first anesthesia. ECG (II): electrocardiogram in lead II, EtCO_2_: end‐tidal carbon dioxide, ABP: arterial blood pressure. (a) Preinduction. (b) Postinduction (1). (c) Postinduction (2). (d) Prone (1 min). (e) Prone (9 min).

**Figure:**
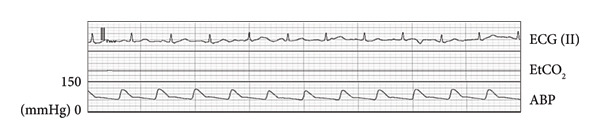
(a)

**Figure:**
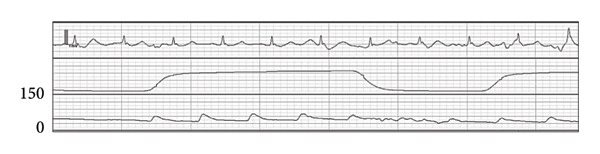
(b)

**Figure:**
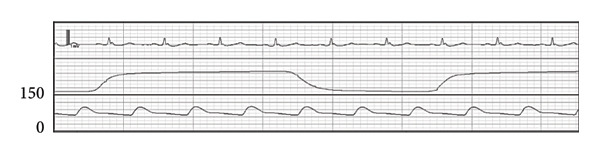
(c)

**Figure:**
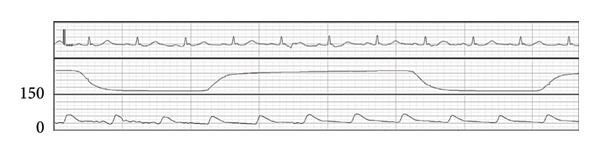
(d)

**Figure:**
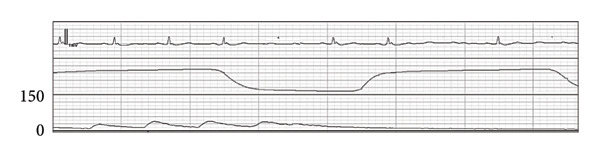
(e)

After transitioning to the prone position, the patient gradually developed hypotension (Figure 2(d)). Nine minutes after the position change, pulseless electrical activity (PEA) occurred (Figure 2(e)), and the patient did not respond to phenylephrine administration. Propofol and remifentanil were immediately discontinued, and the patient was promptly repositioned from the prone to supine position. Cardiopulmonary resuscitation (CPR) was initiated, including chest compressions and administration of approximately 1000 mL of crystalloid solution and adrenaline. These interventions successfully restored the spontaneous circulation.

Approximately 10 min after adrenaline administration, the patient again showed a tendency toward hypotension, prompting the initiation of continuous infusions of noradrenaline and dopamine. Following the start of catecholamine administration, hypotension resolved. The sBP stabilized at approximately ≥ 85 mmHg. Approximately 30 min after the return of spontaneous circulation, the patient regained spontaneous breathing and was responsive to commands. The ventilator was subsequently removed and the patient was extubated. The patient was then transferred to the intensive care unit (ICU) for comprehensive management. As a result, the planned surgical procedure was canceled.

We immediately investigated the cause of the patient’s progression to PEA after induction of general anesthesia. Given the absence of skin findings or bronchospasm, anaphylactic shock was deemed unlikely, and a cardiogenic cause was suspected. Blood gas analysis showed no acid–base imbalance or electrolyte abnormalities such as hyperkalemia, making arrhythmic cardiac arrest unlikely. UCG performed in the ICU revealed no significant changes compared with the preoperative findings. Additionally, no new LV motion abnormalities were observed, making ischemic heart disease unlikely. However, coronary angiography was performed as a precaution. The results revealed no significant stenosis requiring treatment. These findings were indicative of circulatory suppression caused by a general anesthetic agent (propofol) being the primary factor contributing to PEA. Fortunately, no apparent complications related to cardiac arrest, such as neurological sequelae or new ischemic organ dysfunction, were observed.

Given the significant risks associated with surgical treatment under general anesthesia, we strongly recommend alternative symptom management strategies for lumbar spinal canal stenosis, such as pharmacological therapy and nerve blocks. However, the patient expressed a strong preference for surgical intervention despite being fully informed of the potential risks of general anesthesia. A multidisciplinary conference involving orthopedic surgeons and anesthesiologists was convened. It was concluded that the previous episode of PEA was likely caused by circulatory suppression induced by the anesthetic agents. Therefore, the use of anesthetic agents with minimal circulatory depressive effects could potentially allow for safer anesthetic management during surgery. After providing the patient with a detailed explanation of the associated risks, a surgical treatment plan was developed.

### 2.2. Second Anesthesia Progress (Figure 3)

For the second anesthetic approach, remimazolam, which is characterized by minimal circulatory suppression, was selected. To mitigate preoperative dehydration, ultrafiltration during hemodialysis on the day before surgery was limited to the dry weight plus 1 kg.

**Figure 3:**
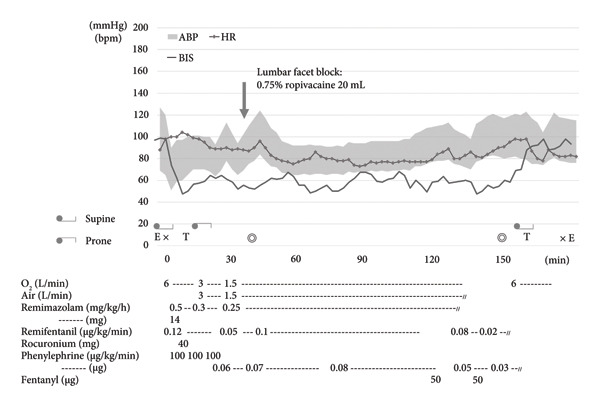
Second anesthesia progress. ABP: arterial blood pressure, HR: heart rate, BIS: bispectral index, E: admission to or discharge from the operating room, X: start or end of anesthesia, T: tracheal intubation or extubation, ◎: start or end of surgery.

As in the first anesthesia procedure, the patient entered the operating room without premedication, and an arterial line was secured in the right radial artery prior to anesthesia induction. After initiating ECG, SpO_2_, and ABP monitoring, general anesthesia was induced via a bolus injection of 14 mg of remimazolam, followed by continuous infusion at 0.5 mg/kg/h, remifentanil at 0.12 μg/kg/min, and 40 mg of rocuronium. Hypotension during anesthesia induction and prior to repositioning was significantly less pronounced than that during the first anesthesia, and the hemodynamics were generally stabilized. Hemodynamic stability was also maintained after repositioning in the prone position.

**Figure 4:**
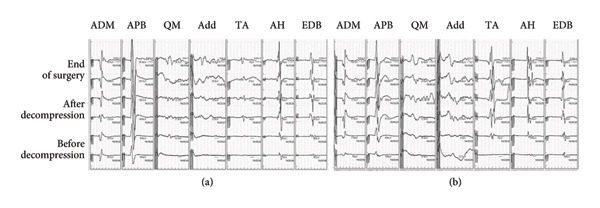
MEP during the second anesthesia. MEP: motor evoked potential, ADM: abductor digiti minimi, APB: abductor pollicis brevis, QM: quadriceps muscle, Add: adductor muscle, TA: tibialis anterior, AH: abductor hallucis, EDB: extensor digitorum brevis.

During surgery, anesthesia was maintained with remimazolam at 0.25 mg/kg/h and remifentanil at 0.1 μg/kg/min, with phenylephrine continuously infused at 0.03–0.08 μg/kg/min as circulatory support. To reduce the required dose of general anesthetics and mitigate anesthetic‐induced circulatory depression, a lumbar facet block was performed prior to the start of surgery using 20 mL of 0.75% ropivacaine. Throughout the surgery, hemodynamic parameters remained highly stable, enabling the smooth and eventful progression of the procedure. Intraoperative MEP monitoring was successfully performed. Before decompression of spinal canal, the amplitude of MEPs in both lower extremities was limited. However, after decompression, a significant increase in amplitude was observed. The surgery then proceeded without any MEP changes indicative of neurological impairment, such as prolonged latency or reduced amplitude (Figure 4).

The duration of the procedures was 1 h and 51 min, with total anesthesia times of 3 h and 1 min, respectively. The estimated blood loss was 80 mL, and the total infusion volume was 662 mL.

## 3. Discussion

Taken together, the results indicate that the primary causes of cardiac arrest (PEA) following first general anesthesia were as follows: (1) circulatory depression caused by general anesthetics; (2) hypovolemia; and (3) hypotension associated with the prone position.

1. It has been reported that propofol shows no significant difference in pharmacokinetics and pharmacodynamics between patients with ESRD and healthy individuals, and that TCI can be administered in a similar manner [9]. However, patients with ESRD are at extremely high perioperative risk, with a reported mortality rate of approximately 4% [10, 11]. Regarding the choice of anesthetic agents, CHO et al. [11] have suggested that propofol anesthesia is associated with a lower incidence of cardiovascular events and better outcomes than volatile anesthesia in ESRD patients. However, because propofol reduces myocardial contractility and vascular resistance, it tends to cause hypotension. This effect is particularly pronounced in ESRD patients, who are more prone to intraoperative hypotension and require greater amounts of vasopressors [12]. In patients with dialysis difficulty, such as in the present case, impaired neurovascular reactivity necessitates even more cautious hemodynamic management of propofol‐induced hypotension. Specifically, lowering the TCI and considering the concomitant use of ketamine to reduce the required dose of propofol would be appropriate strategies.
 Remimazolam is known to cause less circulatory depression than propofol [5]. Hasegawa et al. [6] investigated autonomic nervous system function through heart rate variability analysis and reported that remimazolam preserved the balance between sympathetic and parasympathetic activities. Additionally, using rat myocardial RNA sequencing, Yoshikawa et al. [7] found that remimazolam does not exert a direct negative inotropic effect on myocardial cells, as these cells lack the γ subunit of the GABA_A_ receptor. In other words, while both remimazolam and propofol decreased peripheral vascular resistance, the impact of remimazolam on cardiac function likely contributed to the less pronounced reduction in blood pressure. This is consistent with the present case, in which hypotension during propofol anesthesia could not be managed with phenylephrine alone, whereas circulatory stability was achieved with only a small dose of phenylephrine during remimazolam anesthesia.
 The maintenance dose of remimazolam is influenced by factors such as hepatic function and age. Previously, we have reported that the required maintenance dose could be approximated as “1.17 − (age × 0.011) mg/kg/hr” [13]. In the present case, the maintenance dose was 0.25 mg/kg/hr, considerably lower than the estimated value of 0.41 mg/kg/hr. It should be noted that a facet block was used combined with general anesthesia, which may have reduced surgical stress and, together with the systemic effects of the local anesthetic, contributed to the reduced required dose of remimazolam. Furthermore, the reduced remimazolam dose may have had a favorable effect by limiting circulatory depression to a mild degree.
2. Prior to the first anesthesia session, fluid was removed to achieve dry weight. During CPR, approximately 1000 mL of fluid was administered, leading to circulatory recovery. Based on this observation, fluid removal was adjusted to the dry weight plus 1 kg during dialysis before the second anesthesia.
 For dialysis patients, performing dialysis immediately before surgery is generally preferred to optimize the electrolyte and acid–base balance. However, because of the bleeding risks associated with anticoagulant use during dialysis, preoperative dialysis is often performed the day before surgery as a safer alternative [14, 15]. Nevertheless, there is no established gold standard regarding the optimal volume of fluid to be removed during preoperative dialysis. In patients with severe atherosclerotic disease and high circulatory risk, as in this case, reducing the volume of fluid to be removed during preoperative dialysis may be a meaningful and prudent approach.
3. The prone position during general anesthesia is known to increase the risk of hypotension and cardiac arrest due to reduced venous return caused by elevated intra‐abdominal pressure [16]. This risk is particularly pronounced in patients with hypovolemia or impaired cardiac function. Kim et al. [17] investigated hypotension during prone position surgery under remimazolam and propofol anesthesia. Compared with propofol, they found that remimazolam was superior in maintaining blood pressure within 10 min of adopting the prone position and required lower doses of ephedrine. These findings support the fact that mitigating preoperative fluid removal and using remimazolam anesthesia in this case helped avoid severe circulatory suppression.

In this case, the administration of general anesthesia posed a significant challenge due to a history of profound hemodynamic suppression leading to cardiac arrest under propofol anesthesia. Notably, the use of remimazolam enabled the uneventful completion of the surgical procedure while maintaining hemodynamic stability. This case adds to the growing body of evidence [5–7] suggesting that remimazolam induces less hemodynamic depression compared with propofol, highlighting its potential advantage in patients at high risk of cardiovascular instability.

**Nomenclature**
ESRD: End‐stage renal disease
DM: Diabetes mellitus
ECG: Electrocardiography
UCG: Ultrasoundcardiography
sBP: Systolic blood pressure
ABP: Invasive arterial blood pressure
PAF: Paroxysmal atrial fibrillation
LV: Left ventricle
LAVI: Left atrial volume index
BIS: Bispectral index
TCI: Target‐controlled infusion
SpO_2_
: Percutaneous oxygen saturation
PEA: Pulseless electrical activity
CPR: Cardiopulmonary resuscitation
RNA: Ribonucleic acid
GABA: Gamma‐amino butyric acid

## Consent

Written informed consent was obtained from the patient for publication of this case report.

## Disclosure

All the authors have read and approved the final version of this manuscript.

## Conflicts of Interest

The authors declare no conflicts of interest.

## Author Contributions

Toshihiro Takeda and Koji Okano prepared the manuscript. Yuichi Ogino supervised and edited the manuscript. All authors have contributed to the editorial changes in the manuscript.

## Funding

No funding was received for this study.

## Data Availability

The data that support the findings of this study are available on request from the corresponding author. The data are not publicly available due to privacy or ethical restrictions.
